# Nuclear roles for cilia-associated proteins

**DOI:** 10.1186/s13630-017-0052-x

**Published:** 2017-05-25

**Authors:** Tristan D. McClure-Begley, Michael W. Klymkowsky

**Affiliations:** Molecular, Cellular and Developmental Biology, University of Colorado Boulder, Boulder, CO 80309 USA

## Abstract

Cilia appear to be derived, evolutionarily, from structures present in the ancestral (pre-ciliary) eukaryote, such as microtubule-based vesicle trafficking and chromosome segregation systems. Experimental observations suggest that the ciliary gate, the molecular complex that mediates the selective molecular movement between cytoplasmic and ciliary compartments, shares features with nuclear pores. Our hypothesis is that this shared transport machinery is at least partially responsible for the observation that a number of ciliary and ciliogenesis-associated proteins are found within nuclei where they play roles in the regulation of gene expression, DNA repair, and nuclear import and export. Recognizing the potential for such nuclear roles is critical when considering the phenotypic effects that arise from the mutational modification of ciliary proteins.

**Electronic supplementary material:**

The online version of this article (doi:10.1186/s13630-017-0052-x) contains supplementary material, which is available to authorized users.

## Introduction

Cilia, whether immotile (primary) or motile, flagella (on sperm), or the cilia-based structures within photoreceptor cells, represent a distinct cytoplasmic and plasma membrane domain [26]. As has been reviewed recently, mutations in cilia- or ciliogenesis-associated proteins can lead to a range of human pathologies, known collectively as ciliopathies [8, 26, 31, 49, 64, 76, 86, 88, 91, 94]. In addition to their well appreciated cilia- and ciliogenesis-associated roles, it is now increasingly apparent that cilia-associated proteins have non-ciliary, cytoplasmic roles, described in a number of recent reviews [87, 93, 102]. For example, it is well established that cilia and cilia-associated proteins are involved in Hedgehog, TGF-β, Wnt, PDGFRα, Notch, and Hippo inter-cellular signaling systems [12, 31, 36, 62, 65, 86]. That said, cilia themselves may not be strictly necessary for a number of these signaling pathways. For example, myeloid and lymphoid cells reportedly lack cilia (primary or motile) yet express cilia-associated proteins [24] and support Hedgehog signaling [16, 93]. Similarly, in the zebrafish, Huang and Schier [37] found that genetic abolition of maternal and zygotic cilia formation left Wnt signaling intact while dysregulating hedgehog signaling.

In addition to their signaling functions, there is clear evidence for the role of a number of cilia-associated proteins in DNA damage repair [4, 102]. In this review, we highlight the observation that a number of cilia-associated proteins have a nuclear presence, and in some cases, functional nuclear roles. We consider the possibility that the nuclear functions of cilia-associated proteins, arising in part from their evolutionary ancestry, can contribute to ciliopathic phenotypes through both developmentally specific and more generic mechanisms.

## Implications of the evolutionary origin of cilia

While speculating about processes that occurred billions of years ago can be problematic, there is a clear consensus that the evolution of cilia began in an ancestor that contained a nucleus, with nuclear pores mediating molecular movement across the nuclear envelope and microtubule-based systems involved in chromosome segregation [10, 27, 58, 59, 70]. Entry into the ciliary domain is regulated through a distinct “transition zone” between non-ciliary cytoplasmic and plasma membrane domains. The details of this ciliary gate are beginning to be resolved [17, 39]. Resendes et al. [73] reported that the basal body protein centrin-2 (Cetn2) interacts with the nucleoporin (Nup) 107–160 complex, an integral component of the nuclear pore. As with the yeast homolog Cdc31 [25, 74], Cetn2 is an integral component of the nuclear pore. The observation that nuclear transport proteins and nuclear localization-like sequences are involved in ciliary transport [19, 38, 83] and that blocking nuclear transport also blocks ciliary import [43, 82] suggests that nuclear pore/transport proteins were co-opted during ciliary evolution. That said, Breslow et al. [7] failed to find Nup localization or a functional role for Nups in the passive permeability barrier of primary (immotile) cilia. It is clear that that this ciliary permeability barrier/transport system, which we refer to as the ciliary gate, is structurally and functionally distinct from the nuclear pore complex. In their studies of various motile cilia in the developing *Xenopus* embryo and cultured human retina pigment epithelial (RPE) cells, del Viso et al. [17] found evidence that the association of the inner ring (of the nuclear pore complex) Nups 188 and 93, but not other Nups examined (e.g., Nup63), are required for the formation of motile cilia in a range of cell types (the authors did not examine immotile, primary cilia). Their structural studies localized Nups 188/93 to the cilia base region, while 3D super-resolution microscopy in RPE cells revealed “the presence of two discrete structures, each composed of dozens of individual localization clusters that could not be resolved by confocal microscopy.” The overall distribution of cilia-associated Nup188/93 is distinctly different from the distribution of these proteins at the nuclear pore.

In the light of these studies, there is a general possibility that a subset of cilia-associated proteins contain localization/interaction sequences that can be recognized by cilia-associated structures, such as the Nups found in the pericentriolar material (PCM) region of the ciliary base, as well as components of the nuclear pore complex. The association of RAN complex components, important in active transport through nuclear pores [61], in the transition zone of cilia supports this model [19, 21]. RAN appears to be involved in “controlling injections of IFT proteins into the flagellar compartment” and “is, therefore, crucial to ciliogenesis” [53]. Similarly, transport of Kif17 [19, 29], retinitis pigmentosa 2 protein (RP2) [38], and Gli2 [89] into cilia is reported to be importin-β2, but not importin-α/β1, dependent (in the case of Gli2).

As examples, both the ciliary axoneme-associated radial spoke protein 3 (RSPH3–OMIM: 615876) [100] and the pericentriolar and ciliary basal body-associated protein pericentrin (PCNT: OMIM: 605925) [51] have been found to contain functional nuclear localization (NLS) and nuclear exclusion (NES) sequences. Similarly parafusin, a signaling scaffolding protein, has been found localized to the base of primary cilia in a variety of mammalian cell types and within the nuclei of fibroblasts [77]. The question arises then how common is it to find cilia-associated proteins in nuclei and what roles, if any, do they play there?

## Characterizing cilia-associated proteins

Eukaryotic cilia are complex organelles composed of modified (double and triplet) microtubules and various associated proteins. Ishikawa et al. [41] identified 195 polypeptides in primary cilia, of which ~75% appeared to be present in motile cilia as well. Proximity labeling studies [57] have identified over 370 cilia-associated proteins. Boldt et al. [5] used an affinity proteomic-based study to identify 1319 cilia-associated proteins, 4905 inter-polypeptide interactions, and 52 molecular complexes. Such interaction complexity highlights the heterogeneous nature of ciliary functions in specific cell types and the likelihood that multiple evolutionary events along the vertebrate lineage have led to multiple functions for many of these proteins.

It is important to consider, however, that just because a protein is found associated with other ciliary components does not mean that its primary or sole function is ciliary. Many proteins are poorly studied, apparently for largely historical rather than functional criteria; Pandey et al. [68] coined the term “ignorome” for such proteins (and the genes that encode them). A dramatic example of the difficulty in defining gene product functions (one basis for the ignorome) is illustrated by the studies of Hutchinson et al. [40] who produced a minimal bacterial genome (JCV-syn3.0) containing 473 genes. Analysis of JCV-syn3.0 revealed an ignorome of 149 genes of unknown function (~32% of the total genome); these are genes required to produce a viable organism. At the same time, there is an increasing awareness that a number of proteins, originally identified as structural or functional components of one cellular system, can have quite distinct functions in another; they are what we might term “multi-taskers.” An example of such multitasking proteins is the catenins. Originally identified in the context of cadherin-based cell adhesion junctions [67], catenins were subsequently recognized to play important roles in the extracellular signaling-mediated regulation of gene expression [47, 54]. In this light, Kustatscher and Rappsilber [48] suggested that proteins have a certain degree of “fuzzy” distribution within cells. This notion, strengthened by proteomic analyses of subcellular compartments and organelle fractionation, indicates that stochastic intracellular movements are evolutionarily advantageous, as they increase the interaction potential of multitasker proteins. Contextually advantageous interactions are eventually “captured” by positive selection. This may well be the case for ciliary proteins in the nucleus, where their shared functions within related structures put them in position to acquire novel roles under specific circumstances.

**Fig. 1:**
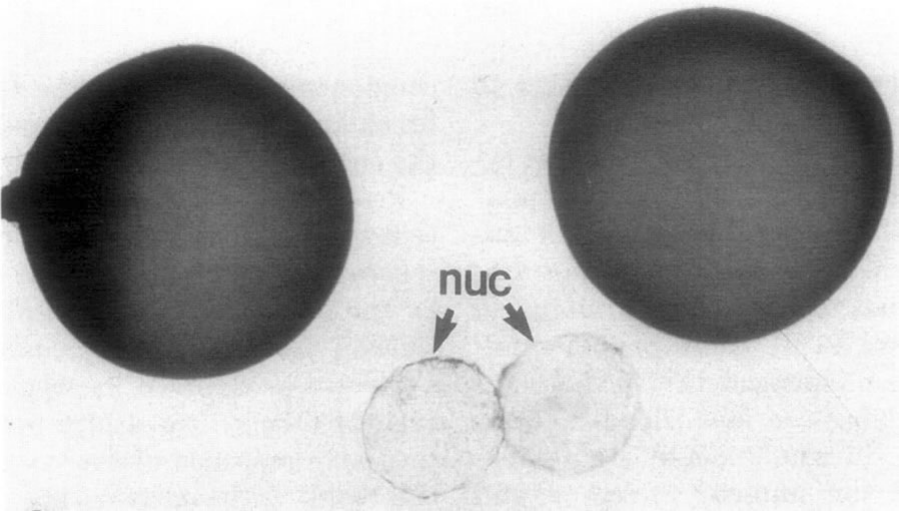
Isolated Xenopus germinal vesicles (image from [46]); each oocyte has a diameter of ~1 mm

One approach to defining the extent to which cilia-associated proteins are localized to nuclei involves mass spectrometric–proteomic studies of isolated nuclei. There are often technical obstacles to overcome in such an analysis, including the possibility of protein leakage and redistribution during nuclear isolation (see [55]). In their study Wühr et al [99] exploited unique aspects of the *Xenopus laevis* oocyte nucleus (the germinal vesicle), specifically its large size (~400 μm in diameter) and the ability to isolate intact germinal vesicles rapidly from late stage oocytes (Fig. 1) [46]. At the same time, oocytes are not known to express cilia of any kind, which limits the analysis to those proteins expressed during later stages of oogenesis. Wühr et al. characterized proteins based on their “relative nuclear concentration” (RNC), defined as the ratio of concentrations in the nucleus to the concentrations in nucleus plus the cytoplasm“; the RNC ranges from 1 for a totally nuclear protein to 0 for a completely cytoplasmic (non-nuclear) protein. Of course polypeptides that reside in the nucleus are synthesized in the cytoplasm, nuclear isolation is not completely “clean” (even with isolated germinal vesicles), and a number of proteins are known to shuttle between nuclear and cytoplasmic compartments, so most RNC values are intermediate between 0 and 1. Nevertheless, it is possible to distinguish classes of proteins that appear to be excluded from nuclei and confined to the cytoplasm (value of RNC <0.1) from those that have a significant nuclear localization signal (RNC >0.35).

To estimate the number of cilia-associated proteins localized to nuclei, we used the Wühr et al. dataset together with the “gold standard” set of ~300 cilia-associated polypeptides described by van Dam et al. [92]^1^; to this database, we added polypeptides known to be involved in ciliary function, i.e., C2orf59 [42], Cetn2 [81, 90, 101], and EFHC1 and EFHC2 (see [103]), for a total of 307 polypeptides. Of these 307 “gold standard” ciliary proteins, Wühr et al. report data on 118; of these, 30 (~25%) have RNC values of >0.35 (Table 1) and so are expected to have a significant nuclear presence. Other 29 polypeptides (~24%) have RNC values between 0.35 and 0.1 and may well be able to enter the nuclei. Within subgroups of related proteins, such as the interflagellar transport polypeptides (IFTs), most appear exclusively cytoplasmic, but a few (e.g., IFT27) (Table 1) seem to be present at significant levels within the germinal vesicle. Similarly, the radial spoke protein RSPH4A (OMIM: 612647) has an RNC value of 0.9, higher than the known transcription factor SOX13 (0.84), suggesting that RSPH4A is strongly concentrated in the germinal vesicle. In contrast, β-catenin (CTNNB1), which is well known to be able to enter the nuclei in response to canonical Wnt signaling (see [54]), appears to be exclusively cytoplasmic (RNC = 0.031) in the oocyte.

**Table 1:** Ciliary proteins with a nuclear presence

Nuclear + cytoplasmic >0.2	Ambiguous between 0.2 and 0.1	Cytoplasmic >0.1
**ARL3**	ADP-ribosylation factor-like protein 3	**RNC** **=** **0.51 (nuc** **+** **cyto)**
	Interactor with RP2 and PDEδ. Involved in trafficking of vesicles from the Golgi to the cilium, especially; farnesylated cargo in association with PDEδ and RPGR; and myristoylated cargo in association with UNC119 and RP2	
**ARL6**	ADP-ribosylation factor-like protein 6	**RNC** **=** **0.49 (nuc** **+** **cyto)**
	Mutated in Bardet–Biedl syndrome (type 3) and retinitis pigmentosa (type 55). At the ciliary gate, it regulates Wnt signaling. Functions with BBSome to coat proteins for trafficking to the cilium basal body, cilium, cytosol, transition zone	
**ATXN10**	Ataxin 10	**RNC** **=** **0.48 (nuc** **+** **cyto)**
	Mutated in 1 NPHP family (splice-site mutation). Forms complex with NPHP5 and 6 at the basal body	
**C21orf59** ^a^	Jaffe et al. [42] c21orf59/kurly controls both cilia motility and polarization	**RNS** **=** **0.48 (nuc** **+** **cyto)**
**C8orf37**	Mutated in cone–rod dystrophy (type 16) and retinitis pigmentosa (type 64). Localizes to basal body in cultured RPE cells and Basal body and ciliary rootlet in mouse photoreceptors Basal body, ciliary root	**RNS** **=** **0.36 (nuc** **+** **cyto)**
**CETN2** ^a^	Centrin-2	**RNS** **=** **0.55/0.58 (nuc** **+** **cyto)**
**CETN3** ^a^	Centrin-3	**RNS** **=** **0.33 (nuc** **+** **cyto)**
**CTNNB1**	Catenin beta-1	**RNS** **=** **0.031 (cytoplasmic** ^a^ **)**
	Involved in regulation of PKD1 and PKD2 expression. Nek2 substrate involved in centrosome separation, along with rootletin (CROCC). Facilitator of canonical Wnt signalling pathway. Many links between cilia and Wnt signaling centrosome	
**DNAL1**	Dynein light chain 1, axonemal	**RNS** **=** **0.49 (nuc** **+** **cyto)**
	Mutated in primary ciliary dyskinesia (type 16) Component of outer dynein arms. Axoneme	
**DPCD**	Deleted in primary ciliary dyskinesia	**RNS** **=** **0.29 (nuc** **+** **cyto)**
	Deleted in a mouse model of primary ciliary dyskinesia	
**EFHC2** ^a^	EF-hand domain-containing family member C2	**RNS** **=** **0.47 (nuc** **+** **cyto)**
**HEATR2**	HEAT repeat-containing protein 2	**RNS** **=** **0.63 (nuc** **+** **cyto)**
	Mutation linked to premature centromere division (PCD), presumably involved in dynein arm transport or assembly	
**HSPA8**	Heat shock cognate 71 kDa protein	**RNS** **=** **0.49 (nuc** **+** **cyto)**
	Chaperone of the IFT together with DnajB6	
**HSPB11**	Heat shock protein beta-11	**RNS** **=** **0.48 (nuc** **+** **cyto)**
	IFT25, part of IFT-B complex. Forms a complex with IFT27. External submission. Cilium, IFT	
**IFT27**	Intraflagellar transport protein 27	**RNS** **=** **0.45 (nuc** **+** **cyto)**
	Component of IFT complex B. Rab-like small G protein basal body, cilium, IFT	
**KIF17**	Kinesin-like protein KIF17	**RNS** **=** **0.21 (nuc** **+** **cyto)**
	Kinesin 2 motor, active in antergrade IFT ciliary tip	
**LRRC6**	Protein TILB homolog	**RNS** **=** **0.34 (nuc** **+** **cyto)**
	Essential for proper axonemal assembly of inner and outer dynein arms, causes PCD. Cilium	
**MAPRE1 (EB1)**	MT-associated protein RP/EB family 1	**RNS** **=** **0.38 (nuc** **+** **cyto)**
	MT plus-end-tracking protein. promotes ciliogenesis. Centrosome, golgi	
**MNS1**	Meiosis-specific nuclear structural protein 1	**RNS** **=** **0.37 (nuc** **+** **cyto)**
	Mns1−/− mice have short, immotile sperm flagella, situs defects, and hydrocephalus. Protein localized along flagellum. Knockdown in IMCD3 and 3T3 cells causes Hh signaling defects axoneme	
**NME7**	Nucleoside diphosphate kinase 7	**RNS** **=** **0.21 (nuc** **+** **cyto)**
	IFT transport and signaling defects after knockdown	
**NME8**	Thioredoxin domain-containing protein 3	**RNS** **=** **0.39 (nuc** **+** **cyto)**
	The sea urchin ortholog of this gene encodes a component of sperm outer dynein arms, and the protein is implicated in ciliary function. Mutations in this gene are implicated in primary ciliary dyskinesia type 6	
**NPHP1**	Nephrocystin-1	**RNS** **=** **0.36 (nuc** **+** **cyto)**
	Known ciliopathy gene JBTS4, NPHP1, SLS1 transition zone	
**NUP214**	Nuclear pore complex protein Nup214	**RNS** **=** **0.45 (nuc** **+** **cyto)**
	Part of ciliary pore complex transition zone	
**NUP35**	Nucleoporin NUP35	**RNS** **=** **0.47 (nuc** **+** **cyto)**
	Nucleoporin 35 kDa, part of ciliary pore complex transition zone	
**NUP37**	Nucleoporin NUP37	**RNS** **=** **0.56 (nuc** **+** **cyto)**
	Part of ciliary pore complex transition zone	
**NUP62**	Nuclear pore glycoprotein p62	**RNS** **=** **0.38 (nuc** **+** **cyto)**
	Part of ciliary pore complex transition zone	
**NUP93**	Nuclear pore complex protein Nup93	**RNS − 0.45 (nuc** **+** **cyto)**
	Part of ciliary pore complex transition zone	
**OCRL**	Oculocerebrorenal syndrome of Lowe	**RNS** **=** **0.24 (nuc** **+** **cyto)**
	Linked to Lowe Syndrome. Involved in assembly of primary cilia, involved in Rab8n-dependent protein trafficking to the cilium	
**ORC1**	Origin recognition complex subunit 1	**RNS** **=** **0.23 (nuc** **+** **cyto**)
	Linked to Meier–Gorlin syndrome (MGS), a disorder conferring microcephaly, primordial dwarfism, underdeveloped ears, and skeletal abnormalities. knockdown affects ciliogenesis and Hh signaling	
**PAFAH1B1**	Platelet-activating factor acetylhydrolase IB alpha	**RNS** **=** **0.28 (nuc** **+** **cyto)**
	LIS1 Mammalian Lis1 localizes to motile cilia in trachea and oviduct, but is absent from non-motile primary cilia axoneme	
**PDE6D**	cGMP 3′,5′-cyclic phosphodiesterase subunit delta	**RNS** **=** **0.40 (nuc** **+** **cyto)p**
	Part of the ARL13B, INPP5E, and CEP164 network	
**PLK1**	Serine/threonine-protein kinase PLK1	**RNS** **=** **0.39 (nuc** **+** **cyto)**
	Localizes to TZ and induces phosphorylation of NPHP1 transition zone	
**RABL5**	Rab-like protein 5	**RNS** **=** **0.33 (nuc** **+** **cyto)**
	IFT22, component of IFT complex B cilium, IFT	
**RILPL2**	RILP-like protein 2	**RNS** **=** **0.50 (nuc** **+** **cyto)**
	Rab effector. Regulates cilium membrane content. Cilium, basal body	
**RSPH4A**	Radial spoke head protein 4 homolog A	**RNS** **=** **0.90 (most nuclear)**
	Defective Hh signaling but no structural cilia defects after knockdown. Mutations in RSPH4A cause primary ciliary dyskinesia, with typical respiratory features, but without situs abnormalities. These findings suggest that radial spoke proteins are not essential for embryonic nodal ciliary function, which is important in the determination of left–right axis development cilium, axoneme	
**RSPH9**	Radial spoke head protein 9 homolog	**RNS** **=** **0.44 (nuc** **+** **cyto)**
	Mutations in RSPH9 linked to primary ciliary dyskinesia, with typical respiratory features, but without situs abnormalities. These findings suggest that radial spoke proteins are not essential for embryonic nodal ciliary function, which is important in the determination of left–right axis development structural cilia defects of primary cilia after knockdown in 3 different murine cell lines cilium, axoneme	
**SNX10**	Sorting nexin-10 (Fragment)	**RNS** **=** **0.51 (nuc** **+** **cyto)**
	Regulates ciliogenesis Centrosome	
**SSNA1**	Sjoegren syndrome nuclear autoantigen 1	**RNA** **+** **0.50 (nuc** **+** **cyto)**
	siRNA knockdown in 3 mouse cell lines perturbs receptor transport into cilium and HH signaling	
**STK38L**	Serine/threonine-protein kinase 38-like	**RNA** **=** **0.34 (nuc** **+** **cyto)**
	Phosphorylates Rabin8. Mutated in canine retinal degeneration. Cytosol	
**SUFU**	Suppressor of fused homolog	**RNS** **=** **0.24 (nuc** **+** **cyto)**
	Localizes to ciliary tip together with GLI transcription factors. Role in Hh signaling ciliary tip	
**TEKT4**	Tektin-4	**RNS** **=** **0.35 (nuc** **+** **cyto)**
	Axonemal protein required for flagella motility in mouse sperm. Expression reduced in inasthenozoospermic men. Cilium	
**TNPO1**	Transportin-1	**RNS** **=** **0.44 (nuc** **+** **cyto)**
	Importin beta 2. Regulates entry of RP2 and kinesin motor into cilium axoneme	
**TRAPPC3**	Trafficking protein particle complex subunit 3	**RNS** **=** **0.30 (nuc** **+** **cyto).**
	Required for Rabin8 centrosome trafficking and ciliogenesis basal body, centrosome	
**TTK**	Dual specificity protein kinase TTK	**RNS** **=** **0.33 (nuc** **+** **cyto)**
	Negatively regulates ciliogenesis. Centrosome	
**WDR19**	WD repeat-containing protein 19	**RNS** **=** **0.22 (nuc** **+** **cyto)**
	Mutations in WDR19 associated with ciliopathies nephronophthisis (NPHP13), Jeune and Sensenbrenner syndromes (ATD5), IFT complex A component (aka IFT144) cilium, IFT	

Xenopus Proteomic data from Wuhr et al. ciliary proteins (and curator notes) from: http://www.syscilia.org/goldstandard.shtml

^a^Indicates known ciliary protein added to list of ciliary proteins

In a related set of unpublished studies, “nuclear” fractions were prepared from human SH-SY5Y neuroblastoma cells and analyzed by mass spectrometry (Additional file 1); of the 307 “gold standard” ciliary proteins, 93 were found within this nuclear (Table 2) fraction, including 15 that also had a “nuclear presence” in the *Xenopus* germinal vesicle dataset (Additional file 1). Together these data support the hypothesis that a significant percentage of the polypeptides linked to cilia formation, function, and phenotypic defects appear to be able to enter, and potentially play functional roles, in the nucleus. At this point, we describe a few of the best characterized of these nuclear functions.

**Table 2:** Cilia-associated proteins found in SHSY5Y-NUCLEAR dataset

ARF4	ARL3^a^	ARL6^a^	ASAP1	ATXN10^a^	Cetn2^a^
Cetn3	CEP135	CEP250	CEP41	CEP89	CEP97
CP110	CROCC	CTNNB1^b^	DNAH1^a^	DNAH11	DNAH5
DPCD	DPYSL2	DYNLT1	EXOC3	EXOC4	EXOC5
EXOC6	FLNA	GSK3B	HEATR2^a^	HSPA8^a^	HTT
IFT27^a^	IFT52	IFT81	INVS	KIF3A	KIF3C
MLF1	NGFR	NME7	MAPRE1^a^ (EB1)	LZTFL1	NPHP4
NUP214^a^	NUP35^a^	NUP37	NUP62^a^	NUP93^a^	OCRL
ODF2	ORC1	PAFAH1B1	PARD3	PCM1	PDE6 D^a^
PHF17	PKD2	PLK1^a^	RAB11A	RAB23	RAB8A
RAN	RANBP1	RFX3	RP2	SEPT2	SEPT7
SGK196	SNAP25	STK38L	SYNE2	TNPO1^a^	TRAPPC10
TRAPPC3	TRIM32	TRIP11	TTC21B	TTC30A	TTC8
TUBA1C	TUBA4A	TUBB2A	TUBB2B	TUBB3	TUBE1
TUBGCP2	TUBGCP3	TUBGCP4	VDAC3	XPNPEP3	

^a^Indicates also found to have a nuclear presence in the Xenopus dataset (Table 1)

^b^JUP/Plakoglobin also present

## Examples of cilia-associated proteins with known nuclear roles

Nephronophthisis (NPH) is an autosomal recessive cystic kidney disease characterized by inflammation and scarring (fibrosis) that compromises kidney function and leads, over time, to end-stage renal disease [98]. Many of the genes linked to NPH, encoding polypeptides known as nephrocystins or NPHPs, have been found to encode proteins localized to or involved in primary cilia formation or cilia-dependent signaling (both Wnt and Shh) which presumably explains phenotypic effects on other organ systems. Where other organs are involved, the disease is termed NPH-related ciliopathy [98]. Of the NPHP-associated genes, a number have been found to have direct or indirect nuclear roles. For example, **NPHP4** (nephrocystin-2: OMIM 607215) interacts with and inhibits the LATS1 kinase, leading to the nuclear accumulation of the transcriptional co-activators YAP and TAZ, two proteins involved in the HIPPO (hypoxia) signaling system [33]. **NPHP9** (also known as NEK8–OMIM 609799) has been found to interact with TAZ, leading to the nuclear localization of the NPHP9–TAXZ complex [34]. TAZ is normally exported to the cytoplasm through interactions with 14-3-3 proteins; NPHP9 appears to compete with 14-3-3 proteins for TAZ binding. NPHP4 inhibits LATS1 phosphorylation of TAZ, which reduces its affinity for 14-3-3 proteins. NPHP4 also interacts with the ubiquitin ligase JADE1 (another ciliary and nuclear component), an interaction that appears to stabilize JADE1 and lead to its nuclear localization where it acts to destabilize β-catenin and inhibit canonical Wnt signaling [6]. NPHP4 itself appears to be primarily perinuclear. The absence of NPHP4 activity leads to an increase in Wnt signaling and subsequent cyst formation in the kidney [6]. Studies in the zebrafish support this mechanism [9]. **NPHP7** (OMIM: 611498) is a GLI-like zinc finger transcription factor involved in the regulation of mesenchymal–epithelial cell behavior, inhibiting the expression of genes such as Snail1 and Wnt4 [72]. Mutations in NPHP7 have been associated with NPH-related ciliopathy [35]. Morpholino-mediated downregulation of NPHP7 has been found to influence the formation of immotile motile cilia and a number of associated phenotypes, including cystic pronephros and ciliary motility in zebrafish [45, 71], presumably due to effects on gene expression. **NPHP10** (OMIM: 613524) localizes to nuclear foci with a number of DNA damage response proteins, including the centrosomal protein CEP164 (OMIM: 614848) [11].

## Oral-facial-digital 1 (OFD1) syndrome

OFD1 (OMIM: 311200) is one of an array of nine phenotypically similar disorders. OFD1 is an X-linked dominant disease associated with facial malformations as well as cystic kidneys [22]. OFD1 has been linked to the gene OFD1 (previously known as Cxorf5) (OMIM: 300170). In a mouse model OFD1 has been associated with defects in primary cilia formation and left–right axis formation [23]. Subsequent studies indicate that OFD1 has a nuclear presence, and plays a role in chromatin remodeling [32] and in double-stranded DNA break repair, mediated by the TIP60 complex [1].

## Bardet-Biedl Syndrome

Bardet–Biedl syndromes are group of pleiotropic oligogenic ciliopathies [64] that have been linked to (at least) nineteen different genes with potentially etiological roles. Of these, **BBS1, 2, 4, 5, 6, 7, 8,** and **10** have been found to enter the nucleus where they can influence gene expression through interactions with the polycomb group member protein RNF2 (Ring Finger Protein 4: OMIM: 602850) [30]. Both BBS1 (OMIM: 209901) and BBS11 (OMIM: 615988) have been reported to interact with and to alter the activity of NPHP7 [45, 71].

## Centrins and pericentrin

Centrins (Cetn) are calmodulin-like proteins associated with centrosomes (microtubule-organizing centers) and the basal body regions of cilia. Two centrin subclasses have been identified, Cetn2-like and Cetn3-like [14, 28]. In the yeast *Saccharomyces cerevisiae*, there is a single centrin gene (Cdc31); the Cdc31 protein is found localized to nuclear pores in addition to the spindle pole body [74]. Mutations in Cdc31 lead to defects in mRNA export [25]. In vertebrate cells, Cetn2 (OMIM: 300006) has been found associated with nucleoporins and localized to nuclear pores; expression of the Cetn2-binding regions of NUP160 led to a decrease in nuclear export of mRNA and proteins, without obvious effects on protein import [73].

While Cetns have been reported to play a role in cell division in vertebrate cells [75], subsequent studies in chick DT40 cells [15] revealed that null mutations in all three centrin genes had no effect on cell division (although effects on cilia formation were not reported). This made it possible to examine the nuclear functions of centrins, two of which had been previously reported. First, Cetn2 is an integral component of the nucleotide excision repair/xeroderma pigmentosum group C (XPC–RAD23–CETN2) complex [3, 63]. Cetn2-null DT40 cells displayed *no* defects in centrosome formation or cell division but were reported to be hypersensitive to UV irradiation [15]. The nuclear excision repair protein Rad33 appears functionally homologous to Cetn2 [18]. The centrin-associated protein pericentrin (PCNT) (OMIM: 605925) has also been implicated in DNA damage repair [96]. Interactions between pericentrin and microcephalin (MCPH1) have been implicated in primordial dwarfism [60], genome instability, and centrosome amplification [2].

More recently, Cetn2 has been found to be part of the nuclear pore-associated TREX-2 complex (GANP, DSS1, ENY2, PCID2, & CETN2) [13]. TREX-2, in turn, has been reported to interact with the Mediator complex, linking the regulation of gene expression and the export of mRNAs from the nucleus [78, 79]. Our own work in *Xenopus* indicates that Cetn2 associates with sites within the promoter regions of a subset of FGF and FGF receptor genes, regulating their expression and that its morpholino-mediated downregulation leads to defects in mesoderm formation [81].

An interesting point to emerge from studies of Cetn2 is that while standard immunofluorescence microscopy reveals it to be concentrated at centrosomes and basal bodies, cell fractionation studies suggest that more than 90% of centrin is soluble [69] and associated with the xeroderma pigmentous complex (XPC) nucleotide excision repair complex [3, 63], a reminder that proteins can redistribute, often dramatically, upon cell solubilization (see [55]).

**Fig. 2:**
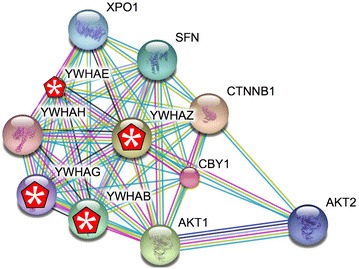
A STRING interaction map for Cby1 (http://string-db.org/cgi/network.pl?taskId=nnIlPEaytPOX) reveals a number of interaction partners. 14-3-3 proteins (YHYAX) marked by * were identified as Cby1-associated proteins in *Xenopus laevis* using an immunoisolation and mass spectrometry analysis (Shi et al. unpublished observations)

**Fig. 3:**
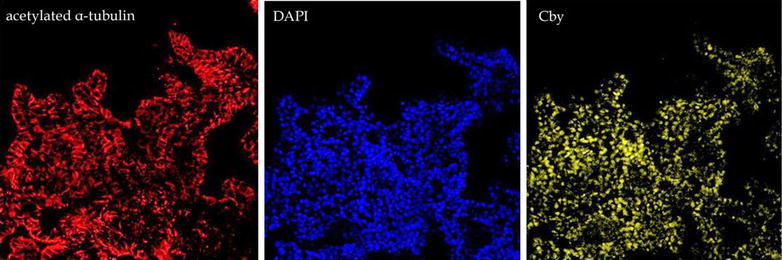
Human iPSC-derived cerebral organoids were stained in whole-mount for acetylated α-tubulin (*lefthand panel*), the DNA marker DAPI (*central panel*), and Cby (*righthand panel*) using a rabbit anti-Cby antibody, supplied by Feng-Qian Li (University of Stonybrook) and described in Li et al. [50]

Chibby (Cby1) (OMIM: 607757) is a small evolutionarily conserved protein associated with basal bodies and involved in ciliogenesis [20]. In humans, there are three Cby-like proteins (Cby, Cby2, and Cby3). In vertebrate cells but not (apparently) in *Drosophila* [20], Cby acts as a negative regulator of β-catenin-mediated Wnt signaling [80, 84, 85].^2^ Cby’s interaction with β-catenin involves 14-3-3 proteins and leads to β-catenin’s export from the nucleus [44, 50]. STRING analysis (Fig. 2) indicates that Cby1 interacts with a number of 14-3-3 (YWHAX) proteins; a conclusion supported by preliminary studies in which Cby-GFP was found to co-precipitate with a number of YWHAX proteins in *Xenopus* (stars in Fig. 2) (McClure-Begley et al. unpublished observations). In human-induced pluripotent stem cell (HiPSC)-derived cerebral organoids, Cby is widely expressed and appears largely nuclear (McClure-Begley et al. in progress) (Fig. 3). Characterization of *cby* ^−*/*−^ mice (in a C57BL/6 background) showed that ~75% died within 2 weeks of birth and were “runted and demonstrated anemia” [95]; those animals that survived displayed a number of cilia-related defects [52]. In *X. laevis*, morpholino-mediated downregulation of Cby led to ciliary defects and abnormal neural crest, central nervous system, and pronephros development [80]. Many, but not all, morphant phenotypes could be ameliorated by the extracellular Wnt inhibitor SFRP2, suggesting that these Cby deficit phenotypes are due to increased Wnt signaling. In this light, Cby morphant effects on Hh components were not rescued by SFRP2, and so presumably represent other (that is, non-canonical Wnt signaling related) processes [80]. A similar behavior appears to be displayed by the 92 kDa nuclear form of Inversin (OMIM: 243305), which has been found associated with β-catenin [66]. Whether, like other β-catenin-interacting proteins [54, 97], Cby1 and Inversin also interact with the paralogous protein plakoglobin (ɣ-catenin or JUP: OMIM: 173325) has not been reported.

## Summary

The challenges in separating the ciliary and cytoplasmic from possible nuclear functions of cilia-associated proteins are similar to those faced in the analysis of the adhesive (cytoplasmic) and gene expression (nuclear) roles of β-catenin and γ-catenin (plakoglobin), which share many interaction partners [97]. The use of cytoplasmically anchored forms allowed us to conclude that γ-catenin’s effects on β-catenin-mediated gene expression were indirect [56]. Given the shared mechanisms acting at nuclear pores and ciliary gates, a similar (or perhaps a more modern) strategy seems necessary to distinguish the ciliary and cytoplasmic from the non-ciliary, i.e., nuclear, functional roles of cilia-associated proteins.

## Additional file

**Additional file 1.** Additional material.

## Abbreviations

OMIM: Online Mendelian Inheritance in Man: https://www.omim.org
RNC: relative nuclear concentration
RPE cells: human retina pigment epithelial cells
NLS: nuclear localization sequence
NES: nuclear exclusion sequence

## Footnotes

1 Website accessible here: http://www.syscilia.org/goldstandard.shtml.

2 Based on a simple BLAST search, there does not appear to a *Caenorhabditis elegans* ortholog of Chibby.
