# Supplementary material for: Nuclear roles for cilia-associated proteins
Source: Cilia. 2017 May 25;6:8. doi: 10.1186/s13630-017-0052-x (PMC5445336; doi:10.1186/s13630-017-0052-x)
Supplement: Supplementary file 1 — Additional file 1. Additional material. [file 13630_2017_52_MOESM1_ESM.pdf]

Supplementary material: SH-SY5Y Neuroblastoma Culture and Differentiation

Tristan McClure-Begley, Chris C. Ebmeier, Douglas A. Chapnick, Kerri E. Ball, & William M. Old*

*Molecular, Cellular & Developmental Biology, University of Colorado Boulder, Boulder Colorado 80309

Description: SH-SY5Y (ATCC CRL-2266) human neuroblastoma cells were grown on 10cm tissue culture dishes under standard conditions (5% CO_2, atmospheric O_2, DMEM:F12 with 10% FBS) until cells were approximately 50% confluent, at which time they were differentiated to a catecholaminergic neuronal phenotype by culturing for 96 hr in 5% FBS with 12nM phorbol-12-myristate acetate (PMA). Cells were then treated with purified cholera toxin A (ChTX; from *Vibrio cholerae*) for 2 hours at 37^oC to stimulate tran slocation of Gαs proteins and establish the utility of subcellular fractionation and quantitative proteomic approaches for detecting dynamic translocation events. After ChTX treatment, cells were rinsed briefly with warm PBS and then frozen by immersion in liquid N2. Cells were stored at -80^oC until fractionation and sample preparation for mass spectrometry.

SH-SY5Y Subcellular Fractionation and Processing for TMT Proteomics: Cell plates were thawed on ice and cells scraped into 1ml Tris-buffered saline (TBS; pH7.5) supplemented with 0.01% Tween-20 and phosphatase and protease inhibitor cocktails (50mM NaF, 1mM Na_3VO_4, 10µg/ml leupeptin and pepstatin, 1mM benzamidine). Crude cell suspensions were initially lysed by trituration of 7 passages through an 18-gauge needle. The resulting lysate was centrifuged at 4°C for 10min at 10,000xg. The supernatant was collected and saved as the cytosolic fraction and the pellet resuspended in 0.2ml of TBS with 0.1% sodium deoxycholate (SDC). This suspension was mixed by rotated at 4°C for 30min, then centrifuged at 16,000xg for 10min. The supernatant was saved as the plasma membrane and vesicular fraction. The final pellet, containing nuclei and insoluble cytoskeletal proteins, was resuspended in 0.2ml SDT buffer (0.1M Tris, pH8.0, 4% sodium docecyl sulfate, 10mM TCEP) and boiled for 10min. Each fraction was denatured, alkylated, and digested according to the FASP

protocol (Wisniewski, Zougman et al. 2009). Eluted peptides were desalted on C18 reverse-phase spin columns, and lyophilized. Dried peptides were resuspended in 0.1M triethylammonium bicarbonate buffer (pH 8.0) and labeled with 10-plex Tandem Mass Tag (TMT) isobaric labels (Viner, Zhang et al. 2009) for multiplexed ratiometric quantitative proteomics. Peptides were labeled in sets corresponding to subcellular fraction, multiplexed within sets by ChTX dose (0, 0.3, 1.0, 3.0, 10.0 µg/ml). Multiplexed peptide samples were fractionated by UPLC on a C18 reverse-phase column at pH10. Samples were analyzed as described by Ting et al (Ting, Rad et al. 2011) Thermo Raw files were processed in Proteome Discoverer (ThermoFisher) for ratiometric protein quantitation. PSMS output files are available on our online proteomics data repository. We established the reproducibility of this method for subcellular fractionation of SH-SY5Y cells by the identification of ChTX dose-dependent translocation of Gαs subunits from the membrane to the cytosolic fraction (supplemental graph 1), and effective ChTX dose was validated by detection of TMT-labeled ChTX peptides using species-specific peptide identification.

Acknowledgements: This work was carried out under a cooperative agreement with DARPA (13-34-RTA-FP-007).

Literature cited:

Ting, L., R. Rad, S. P. Gygi and W. Haas (2011). "MS3 eliminates ratio distortion in isobaric multiplexed quantitative proteomics." Nature methods **8**(11): 937-940.

Viner, R. I., T. Zhang, T. Second and V. Zabrouskov (2009). "Quantification of post-translationally modified peptides of bovine α-crystallin using tandem mass tags and electron transfer dissociation." Journal of proteomics **72**(5): 874-885.

Wisniewski, J. R., A. Zougman and M. Mann (2009). "Combination of FASP and StageTip-based fractionation allows in-depth analysis of the hippocampal membrane proteome." Journal of proteome research **8**(12): 5674-5678.
